# Parental assessment of physical education in the school curriculum: A brief report on the influence of past experiences as students

**DOI:** 10.1371/journal.pone.0219544

**Published:** 2019-07-10

**Authors:** Joaquín Lago-Ballesteros, João Martins, Miguel Ángel González-Valeiro, María A. Fernández-Villarino

**Affiliations:** 1 Faculty of Teacher Training, University of Santiago de Compostela, Lugo, Spain; 2 Pedagogy Laboratory, Faculty of Human Kinetics and Unit for Research and Development in Education and Training, Institute of Education, University of Lisbon, Lisbon, Portugal; 3 Faculty of Physical Education and Sport, Universidade Lusófona, Lisbon, Portugal; 4 Faculty of Sport Sciences and Physical Education, University of Coruña, A Coruña, Spain; 5 Faculty of Education and Sport Sciences, University of Vigo, Pontevedra, Spain; University of Newcastle, AUSTRALIA

## Abstract

The aim of this study was to analyse the relationship between parents' past experience as Physical Education (PE) students and the importance they give to PE within the school curriculum. Parents of 1834 teenagers from Spain and Portugal participated in the study (1834 fathers and 1834 mothers). An 11 item questionnaire was used for data collection. The measures studied were: socio-demographic characteristics, parent´s past experience as PE students, and importance that parents gave to PE in the school curriculum. The results suggest that parents’ past experiences as PE student condition their evaluation of the importance that PE should have in the school curriculum. As the past experience as PE student deteriorated and as age increased, there was an increase in the probability that parents evaluate PE as deserving a less important status in their children's curriculum. These findings can contribute to understanding how the parents' past experiences as PE students seem to partially model the value judgements that they make later in life regarding the importance of the subject.

## Introduction

Global increasing rates of overweight and obesity in children and adolescent population are considered, now and for the future, among the main threats to individuals and communities [1,2]. The relationship of these conditions with cardiovascular disease and type 2 diabetes is well established so their growing prevalence leads to an earlier appearance of these dangerous comorbidities [3], posing a major risk to public health [4] and turning overweight and obesity prevention an imperative.

The etiology of overweight and obesity during childhood and adolescence is multifaceted [5,6], and some of their major determinants are behavioral [7]. Observational studies have shown the association between inadequate dietary habits [8], sedentary behaviors [9] and low physical activity [10] with overweight and obesity in youth. These influences, as behavioral, are potentially modifiable [7] by means of health education programs [11]. In this regard, ecological models have been used to develop comprehensive intervention approaches predominantly at schools [12]. Schools are considered not only as logical sites [13,14] but the ideal place [15] to carry on health education.

When considering the promotion of physical activity at schools, the Comprehensive School Physical Activity Program (CSPAP) conceptual framework has emerged [16] and been rapidly consolidated in research and practice [17]. Physical education is incorporated in this framework as an essential pillar with the goal of providing rich learning opportunities to all students so they acquire the knowledge (i.e., understanding), skills (i.e., competencies), and dispositions (i.e., attitudes, values, self-efficacy beliefs) needed to adopt physically active lifestyles at youth stages and sustain them throughout life [18]. This goal can only be achieved if sufficient instruction is provided but, unfortunately, physical education is only allocated 2–3 teaching hours per week in the secondary education curricula in Spain and Portugal [19]. This time allocation does not meet international established standards for quality physical education at middle and secondary school, as it has been recommended a total of 225 minutes/week [20]. Physical education is compulsory for all students in both countries but, when comparing the share of total teaching time allocated to physical education and that earmarked for other subjects (e.g., mathematics, languages), a lower status of physical education is revealed [21]. However, with regard to pupil assessment, physical education is granted with equal legal status and the educational authorities of Spain and Portugal issue clear recommendations on assessment methods encompassing both formative and summative assessments [19]. Furthermore, physical education teachers have been attributed with a leadership role within the CSPAP framework, as the most logical person to spearhead efforts and coordinate PA promotion [22,23]. In the fulfillment of these responsibilities, PE teachers should involve all the relevant stakeholders in the educational community, and families are one of them. Parent’s collaboration with PE efforts becomes crucial as they can play an important role in their children’s engagement in PA [24–27].

Existing literature has indicated a number of mechanisms to explain the referred parental influence (i.e., genetics, direct modelling, behaviour reward and/or punishment, setting up or elimination of barriers, providing resources for behaviour development) [28–30]. When verifying some of these mechanisms, most research focused on the parental modelling of PA behaviour, resulting in inconclusive evidence [31–33]. According to other studies, the support provided by parents and their beliefs on PA have emerged as important predictors of student engagement in PA [31,33,34]. In this regard, there is evidence that parents are more likely to provide the appropriate support for their children’s PA when they perceive PA as enjoyable and important [34,35]. From a PE pedagogy perspective, past research raised concerns that parents did not always perceive PE as valuable in PA promotion [36]. Gaining parents support has been also perceived as a key challenge by PE teachers to ensure consistency of messages between home and school [37]. Among the barriers that hinder adequate collaboration, a lack of parental knowledge and understanding of healthy lifestyle practices has been highlighted [37,38]. One other study [39] noted that a favourable attitude by parents towards PE is related to a higher level of PA in their children so it would be hypothesized that negative attitudes could also represent a barrier. Thus, the understanding of the processes that lead parents to establish their value judgements about PE is a crucial part of informing future work.

Considering this issue, previous studies have reported on the lasting negative impacts of adverse childhood PE experiences [40,41], but have been limited by methodological weaknesses such as intentional sampling or the use of specific populations. This study aims to analyse the relationship between parents' past experience as PE students with the importance they give to PE within the school curriculum.

## Materials and methods

### Sample

The sample consists of the parents of 1834 teenagers from Spain and Portugal. A total of 1834 fathers and 1834 mothers participated in the study, where 1037 were Portuguese and 797 were Spanish in both cases. For the selection of the participants an intentional non-probabilistic sampling was carried out where 32 secondary schools were chosen, located in urban areas: 6 in the area of Lisbon and 26 in the autonomous region of Galicia. In this selection, schools in different neighbourhoods were chosen to maximise the possibility of achieving a sample consisting of teenagers from different socio-economic levels. Once the target schools were established, the parents of the 3950 teenagers enrolled in them were offered anonymous and voluntary participation. Each teenager was given a large envelope to take home to their parents. Inside the envelopes were two questionnaires and two smaller envelopes (one of each for the father and another for the mother), an explanation sheet with the general aims of the research and the participation rules. After completing the questionnaires, the participants placed them inside the small envelope and sent them back to the school, where they were collected by the teachers and handed over to the research team. Only the envelopes containing the two questionnaires were considered for the study in an attempt to get a balanced distribution of mothers and fathers in the sample.

Prior to the participation in the study, permission was obtained from the directors of the schools. Parents’ consent was given by completing the survey. The study was carried out in accordance with the ethical standards in sport and exercise science [42], and its performance was approved by Portugal’s Faculty of Human Kinetics Council of Ethics and by the Portuguese Ministry of Education.

### Measures

#### Socio-demographic data

Information on the participants was collected in reference to the following demographic variables: sex, age, education level (elementary, secondary, or higher).

### Parents' PA

The parents were asked to report on the type of PA they normally engage in and to specify the frequency and duration of the activity [43,44]. After processing their responses, the participants were classified under the categories of "sufficiently active" or "insufficiently active", depending on whether they fulfilled the main recommendations of the internationally established practice of PA (at least 150 minutes of moderate-intensity physical activity or at least 75 minutes of vigorous-intensity physical activity or an equivalent combination of both intensity levels of activity) [45].

### Past experience as PE students

The questionnaire submitted to the participants included an item where they had to evaluate their past experience as PE students. This item consisted of a likert scale with the following levels: (1) very bad; (2) bad (3); not bad or good; (4) good; (5) very good. When assessing their past experience as physical education students, parents were invited to consider the following elements: teacher’s competence, personality and attitude, obtained benefits (e.g. motor learning, physical condition), social relationships in the classroom, materials, equipment and facilities condition, subject contents, subject and school organization, characteristics of the classes, and any other they judged important.

### Importance of PE in the school curriculum

Finally, the participants were asked to evaluate the importance of PE as a school subject, indicating the conditions under which, in their opinion, it should be included within the curriculum. To do so, they were asked to take a stance in favour of one of the following categories [39]: (1) it should be compulsory and assessable; (2) it should be compulsory but not assessable; (3) it should be elective; (4) it should not be included; (5) DK/Refused.

### Statistical analysis

The analysis of the data was carried out in three stages. First, a descriptive analysis was done for the socio-demographic and nationality identification of the overall sample.

Next, chi-square tests were performed to analyse the parental assessment of past experience as PE student, parents’ support of PE and Parents’ PA according to sex and nationality.

Last of all, a multivariate analysis (binary logistic regression), was performed to jointly explore the relationship of the different independent variables with the dependent variable. A hierarchical backward step-wise regression [46] analysis was used as a modelling strategy. For this analysis the dependent variable was collapsed into two categories (0 = the compulsory and assessable status that PE currently has within the curriculum must be maintained; 1 = PE must be granted a lesser status than the one it currently has within the school curriculum).

The level of statistical significance was set at p<0.05 and all the analyses were performed through version 22.0 of the statistical analysis software package SPSS (Armonk, NY: IBM Corp) for Windows.

## Results

Table 1 shows the socio-demographic characteristics of the parents participating in the study. The demographics of the cohort reflect those of the general population as results showed that mothers were, on average, approximately two years younger than fathers. When comparing parents’ age as a function of nationality, no differences were observed. Regarding level of studies, a higher percentage of higher education studies was found for mothers (31.0%) than for fathers (28.8%), and the same applies to Spanish parents (40.4%) with respect to Portuguese ones (22.6%).

**Table 1:** Socio-demographic characteristics of the parents participating in the study (N = 3668).

Characteristic		Father			Mother		
		Spanish	Portuguese	Total	Spanish	Portuguese	Total
Age (years)		45.0 ± 7,5	44.4 ± 7.8	44.7 ± 7.7	42.8 ± 5.7	42.0 ± 6.6	42.4 ± 6.2
	*n* valid	794	1033	1827	789	1034	1823
	≤29 year	25 (3.1)	20 (1.9)	44 (2.4)	13 (1.6)	12 (1.2)	25 (1.4)
	30–39 year	93 (11.7)	225 (21.8)	318 (17.4)	170 (21.5)	358 (34.6)	528 (29.0)
	40–49 year	497 (62.7)	595 (57.6)	1092 (59.8)	530 (67.2)	560 (54.2)	1090 (59.8)
	50–59 year	162 (20.4)	156 (15.1)	318 (17.4)	74 (9.4)	87 (8.4)	161 (8.8)
	60–69 year	12 (1.5)	25 (2.4)	37 (2.0)	2 (0.3)	14 (1.4)	16 (0.9)
	≥70 year	5 (0.6)	12 (1.2)	17 (0.9)	0	3 (0.3)	3 (0.2)
Education level							
	*n* valid	695	1001	1696	705	1003	1708
	Elementary	268 (38.6)	534 (53.3)	802 (47.3)	243 (34.5)	493 (49.2)	736 (43.1)
	Secondary	157 (22.6)	249 (24.9)	406 (23.9)	167 (23.7)	276 (27.5)	443 (25.9)
	Higher	270 (38.8)	218 (21.8)	488 (28.8)	295 (41.8)	234 (23.3)	529 (31.0)

*Note*. The values in brackets represent the percentage of valid cases.

Table 2 presents basic descriptive statistics for the parental assessment of past experience as PE student, parents’ support of PE and Parents’ PA. Mothers significantly reported better past experiences as PE student than fathers (χ^2^ = 23.70, *p* < .01) and the same occurred for Portuguese parents with respect to Spanish ones (χ^2^ = 23.70, *p* < .01). When analysing parents’ support of PE no differences were found neither between mothers and fathers (χ^2^ = 1.89, *p* = .76) nor between nationalities (χ^2^ = 4.14, *p* = .39). With regard to parents’ PA, no differences were observed between mothers and fathers (χ^2^ = 3.79, *p* = .05) but Spanish parents were more active than Portuguese ones (χ^2^ = 86.30, *p* < .01).

**Table 2:** Descriptive statistics of parental assessment and physical activity (N = 3668).

Variables	Father		Mother		Spanish		Portuguese	
	*n*	%	*n*	%	*n*	%	*n*	%
Past experience as a PE student	1561		1597		1487		1671	
Very bad	76	4.9	39	2.4	78	5.2	37	2.2
Bad	208	13.3	161	10.1	209	14.1	160	9.6
Not bad or good	480	30.7	534	33.4	468	31.5	546	32.7
Good	636	40.7	674	42.2	560	37.7	750	44.9
Very good	161	10.3	189	11.8	172	11.6	178	10.7
Importance of the PE in the school curriculum	1811		1806		1572		2045	
DK/Refused	39	2.2	40	2.2	28	1.8	51	2.5
Not included	17	0.9	10	0.6	9	0.6	18	0.9
Elective	90	5.0	89	4.9	75	4.8	104	5.1
Compulsory and not assessable	656	36.2	650	36.0	583	37.1	723	35.4
Compulsory and assessable	1009	55.7	1017	56.3	877	55.8	1149	56.2
Parents’ PA	1834		1834		1594		2074	
Sufficiently active	654	35.7	711	38.8	728	45.7	637	30.7
Insufficiently active	1180	64.3	1123	61.2	866	54.3	1437	69.3

*Note*. PE = Physical Education, DK = dont know, PA = Physical Activity.

The multivariate binary logistic regression analysis yielded a final model that included only the main effects of past experience as PE student and age (Table 3). As the past experience as PE student deteriorated and as age increased, there was an increase in the probability that parents evaluate PE as deserving a less important status than the one it currently has in their children's curriculum (p<0.01). Neither main effect nor confounding was detected for parents’ PA level so this variable was not retained in the final model.

**Table 3:** Logistic regression model to predict the parental assessment of physical education in the curriculum. (N = 3068).

Variables		*B*	Error	*OR*	*95% CI*	Wald	*p*
Constant		-1.65	0.28	0.19		35.33	<0.001
*Experience as a PE student*						34.43	<0.001
	Very bad	0.80	0.23	2.23	[1.43; 3.49]	12.35	<0.001
	Bad	0.57	0.16	1.77	[1.30; 2.41]	12.96	<0.001
	Not bad or good	0.72	0.13	2.04	[1.57; 2.66]	28.58	<0.001
	Good	0.41	0.13	1.51	[1.17; 1.95]	10.06	0.002
*Age*		0.02	0.01	1.02	[1.01; 1.03]	11.84	0.001

*Note*. PE = Physical Education; OR = *Odds Ratio*; CI = Confidence Interval to *Odds Ratio*.

Finally, when evaluating the proposed regression model, a coefficient of determination of 0.02 was obtained and 58.1% of the parents were classified correctly, with a sensitivity of 14.7% and a specificity of 90.9%. As regards goodness of fit, the Hosmer-Lemeshow test (*χ*^2^ = 10.22; *p* = 0.250) indicates that the model presents an acceptable internal validity.

## Discussion

The purpose of this study was to analyse the relationship between parents' past experience as PE students with the importance they give to PE in the school curriculum. The study results suggest that past experience as PE students seems to partially model the value judgements that parents make later in life regarding the importance of the subject for their children.

Previous studies have identified certain past specific negative experiences (i.e., being the last one chosen for a team) that could lead to the adoption of negative beliefs towards PA and to low levels of participation during adulthood [40,41]. Although this research has adopted a different methodological strategy, based on a global evaluation of parents' past experience as PE students to establish the necessary balance between positive and negative experiences, the results obtained agree with those previously observed, whereby it could be stated that the negative experiences related to PE could determine both their value judgements and the behaviour they adopt in later life stages.

The social support that PE receives has been considered key when defending this discipline, seen as an essential part of the school curriculum, from the reduction plans that have occasionally been suggested, either for financial reasons, or by reducing its importance in comparison to other subjects [47]. When analysing PE's social support among the parents participating in this study, regardless of sex or nationality, approximately 92% consider that PE should be compulsory. This percentage is in keeping with the values reported in existing literature [39,48,49] that indicates that a majority of parents support the need for PE at school. On the other hand, from the results obtained it is worth highlighting that about 40% of those parents who think PE should be maintained as a compulsory subject would reduce its importance in the curriculum, making it non-assessable. This data indicates the need for further studies to deeply analyse the influence of the different PE evaluation practices [50] on the evaluation of parents' past experience as students of the subject, for it is possible that an important part of the negative experiences related to PE have to do specifically with the evaluation procedures used. Likewise, it may be suggested that schools should work on raising the awareness of all the agents belonging to the education community regarding the importance of evaluation as an intrinsic part of PE as it provides information not only about student development, learning and performance but also about the quality of the instruction and programs [51, 52]. Focusing evaluation on progressing towards individual self-established goals regarding healthier lifestyles and the promotion of self-directed learning with regard to the prescription of physical activity may prove useful strategies, for in addition to directly involving the students, these goals and advances could be shared with the rest of the community agents [53].

The influence detected for the age variable, where its increase results in an increase of the probability that parents evaluate PE as deserving a lower status than the one it currently enjoys in the curriculum, is an indication that the reputation of PE improved with time, maybe as a result of the huge effort made in recent decades to highlight the major specific contribution that PE can bring to society [54,55] and, more recently, by the promotion of quality PE classes [56]. The absence of differences with regard to sex or nationality in the evaluation of the importance of PE seems to reveal the international nature of this effort [57] as well as placing emphasis on the promotion of equal access and regular participation [58].

It could be hypothesized that active people would tend to be more sensitive to the importance of PE but the multiple regression analysis showed that Parents’ PA level is not a predictor of Parents’ support of PE in the school curriculum and does not act as a confounding variable nor a modifier of their past experiences as PE student. This finding emphasizes that support for PE and PA are very different constructs. While the main aim of PE in the school curriculum is to develop motor competence and previous literature indicates that motor competence is positively associated with physical activity [59], this relationship seems to be widely unknown for the parents in Spain and Portugal in the light of the present results. As it has been point out, parents did not always perceive PE as valuable in PA promotion [36] but gaining parents support can be considered as a key challenge by PE teachers to ensure consistency of messages between home and school [37]. To increase parents’ awareness of the synergistic relationships among physical education, motor competence, perceived motor competence, physical activity, health-related physical fitness, and obesity, would be an advisable way to gain that support.

In spite of the evidence that this research provides on the influence that the experience provided by PE classes during early stages in life has on the value judgements shown towards the subject during adulthood, certain limitations that could affect the consistency of the results shown here have to be acknowledged. First of all, it needs to be emphasised that while parents were directed to reflect on their experience considering a range of aspects, as this was a single item in the survey, the results cannot draw any inferences about the sources of parents’ values or the balance of positive and negative influences. Secondly, the sample was selected through an intentional non-probabilistic sampling whereby, in spite of the fact that its size is large and that care was taken to choose schools from neighbourhoods with different socio-economic status, the possibility of bias cannot be ruled out. Thirdly, the lack of consideration of family structure and inclusion of two parent families only represents a major limitation and, although two parent family are the most common family arrangement in Spain and Portugal, some valuable data could have been excluded due to these methodological weaknesses. And, fourthly when analysing the quality of the proposed regression model, the low coefficient of determination observed indicates a limited explanatory power, leaving a wide margin for the incorporation of other variables (e.g. PE program contents, ranked comparison with other subjects, perceived contribution of PE toward children’s education, perceived learning in PE)[36,60] that could increase the capacity to explain parents' evaluation of the importance of PE in the school curriculum and, above all, to improve sensitivity, which is the model's main flaw.

As a conclusion, parents generally value PE but their belief that it should be compulsory and an assessable part of the curriculum is varied and associated with their own experiences and knowledge. When implementing a CSPAP, school for parents interventions could be useful to increase parents awareness of current standards for quality PE and the role that PE can play in the promotion of physically active lifestyles. Future research should explore if this kind of interventions could reverse parents’ lack of knowledge or misconceptions and bad past experiences.

## Supporting information

S1 Dataset The data used in the analysis. (SAV)

S1 Supporting Information Spanish questionnaire. (PDF)

S2 Supporting Information Portuguese questionnaire. (PDF)

S3 Supporting Information English questionnaire. (PDF)
