# Supplementary material for: Parental assessment of physical education in the school curriculum: A brief report on the influence of past experiences as students
Source: PLoS One. 2019 Jul 10;14(7):e0219544. doi: 10.1371/journal.pone.0219544 (PMC6619797; doi:10.1371/journal.pone.0219544)
Supplement: S1 Supporting Information — (PDF) [file pone.0219544.s002.pdf]

UNIVERSIDADE DA CORUÑA

Universidade Vigo

Nº.:

CUESTIONARIO PARA LAS/LOS MADRES/PADRES SOBRE LA SALUD, ESTILO DE VIDA Y EDUCACIÓN FÍSICA

Este cuestionario se destina a un estudio sobre la Educación Física, la salud y el estilo de vida. Por eso le pedimos su colaboración. Agradecemos que responda con precisión y sinceridad a las preguntas. Lo que deseamos es saber su opinión.
El cuestionario es anónimo y los datos serán tratados con confidencialidad. Las respuestas son individuales y no hay necesidad de identificarse. La única relación de identificación es la de padres e hijos.
Gracias por cubrir este cuestionario y por participar en esta investigación.

1- DATOS PERSONALES

Este cuestionario fue respondido por:

1. Padre ❑
2. Madre ❑
3. Otro Parentesco ❑ ¿Cuál?_________________________

1. Fecha de nacimiento:

____/____/________
Dia Mes Año

2. Titulación académica y profesión:

2.1. Titulación :

1. Estudios primarios	❑
2. Estudios secundarios	❑
3. Formación profesional	❑
4. Diplomado/licenciado	❑
5. Doctor	❑

2.2. Profesión:_____________________________________

2 - HÁBITOS DE VIDA

3.a. ¿Realiza actividades físico/deportivas **sin ser** en clubs o asociaciones, como por ejemplo: pasear, correr, andar en bicicleta, nadar, jugar al fútbol en la calle, jugar al baloncesto en el parque, jugar al voleibol en el jardín o en la playa, etc.? (**Considere solamente las actividades que practica con una duración superior a 30 minutos por día**)

1. Nunca	❑
2. Menos de una vez por semana	❑
3. Una vez por semana	❑
4. 2 a 3 veces por semana	❑
5. 4 a 6 veces por semana	❑
6. Todos los días	❑

3.b. De acuerdo a lo que contestó anteriormente, ¿cuánto tiempo diría que realiza este tipo de actividad física cada semana con una intensidad moderada o alta? (a punto de finalizar sudando o cansado).

1. Aproximadamente ½ hora	❑
2. Aproximadamente 1 hora	❑
3. Aproximadamente 2 a 3 horas	❑
4. Aproximadamente 4 a 6 horas	❑
5. 7 horas o más	❑

4.a. ¿Practica alguna actividad físico/deportiva en un club o colectivo, bajo la dirección de un entrenador, monitor, instructor, etc.? (**Considere solamente las actividades que practica con una duración superior a 30 minutos por día**)

1. Nunca	❑
2. Menos de una vez por semana	❑
3. Una vez por semana	❑
4. 2 a 3 veces por semana	❑
5. 4 a 6 veces por semana	❑
6. Todos los días	❑

4.b. De acuerdo a lo que contestó anteriormente, ¿cuanto tiempo diría que realiza este tipo de actividad física cada semana con una intensidad moderada o alta? (a punto de finalizar sudando o cansado).

1. Aproximadamente ½ hora	❑
2. Aproximadamente 1 hora	❑
3. Aproximadamente 2 a 3 horas	❑
4. Aproximadamente 4 a 6 horas	❑
5. 7 horas o más	❑

3 – VALORACIÓN DE LA ESCUELA Y DE LA EDUCACIÓN FÍSICA

5. En relación a su vida académica, ¿tuvo la materia de Educación Física? **(Si no tuvo la materia de Educación Física, pase a la pregunta número 8)**

Si ❑ No ❑

6. Tomando como referencia su experiencia personal como alumno, ¿cómo clasifica la Educación Física en la Escuela (o escuelas) a las que asistió?

1. Muy mala	❑
2. Mala	❑
3. Ni mala, ni buena	❑
4. Buena	❑
5. Muy buena	❑

7. Diga la importancia que los factores abajo explicitados tuvieron para la formación de su opinión.

	Importancia	
	Si	No
1. La competencia, personalidad y actitud del profesor	❑	❑
2. Los beneficios adquiridos (aprendizaje motor, condición física, etc.)	❑	❑
3. La convivencia/relación con los compañeros	❑	❑
4. Las condiciones materiales/instalaciones	❑	❑
5. Los contenidos (actividades enseñadas y aprendidas)	❑	❑
6. La organización de la Escuela/Educación Física	❑	❑
7. Las características de las clases	❑	❑
8. Otro<br>¿Cuál? ___________________________________	❑	❑

8. En relación a la existencia de la materia de Educación Física en el currículum escolar **de su hijo/a**, considera que esta disciplina debe **(Escoja la afirmación con la cual esté más de acuerdo)**:

1. Ser obligatoria y sujeta a la evaluación como cualquier otra asignatura	❑
2. Ser obligatoria pero sin que los alumnos sean evaluados	❑
3. Ser optativa	❑
4. No debe existir	❑
5. No tengo opinión sobre este asunto	❑

Gracias por su colaboración
