# Supplementary material for: Parental assessment of physical education in the school curriculum: A brief report on the influence of past experiences as students
Source: PLoS One. 2019 Jul 10;14(7):e0219544. doi: 10.1371/journal.pone.0219544 (PMC6619797; doi:10.1371/journal.pone.0219544)
Supplement: S2 Supporting Information — (PDF) [file pone.0219544.s003.pdf]

REDE EUROAMERICANA DE ACTIVIDADE FÍSICA, EDUCAÇÃO E SAÚDE

Questionário Internacional sobre Educação Física, Saúde e Estilo de Vida

Este questionário destina-se a um estudo sobre a saúde e o estilo de vida. Para isso pedimos a sua colaboração. Agradecemos que responda com precisão e sinceridade às questões. Aquilo que desejamos saber é a sua opinião.

Os dados serão tratados com confidencialidade e não há a necessidade de se identificar, sendo as respostas individuais apenas do conhecimento da equipa de investigadores. A única relação identificada é pais e filhos.

Obrigado por preencher este questionário e por participar nesta investigação.

1 – DADOS PESSOAIS

Este questionário foi respondido por:

1. Pai ❑
2. Mãe ❑
3. Outro Parentesco ❑ Qual?_________________________

1. Data de nascimento:

____/____/________
Dia Meses Ano

2. Habilitação académica e profissão:

2.1. Habilitações:

1. Estudos primários	❑
2. Estudos secundários	❑
3. Formação porfissional	❑
4. Graduado/Licenciado	❑
5. Doutor	❑

2.2. Profissão ________________________________

2 – HÁBITOS DE VIDA

3.a. ¿Realiza atividades físicas e esportivas sem estar em clubes ou associações, como: caminhar, correr, andar de bicicleta, nadar, jogar futebol na rua, jogar basquete no parque, jogar vôlei no jardim ou na praia etc.).? (**Considere apenas as atividades que você pratica com uma duração de mais de 30 minutos por dia**)

1. Nunca	❑
2. Menos de uma vez por semana	❑
3. Uma vez por semana	❑
4. 2 a 3 veces por semana	❑
5. 4 a 6 veces por semana	❑
6. Todos os días	❑

3.b. De acordo com o que você disse anteriormente, quanto tempo você diria que você faz esse tipo de atividade física a cada semana com uma intensidade moderada ou alta? (prestes a acabar suando ou cansado)

1. Aproximadamente ½ hora	❑
2. Aproximadamente 1 hora	❑
3. Aproximadamente 2 a 3 horas	❑
4. Aproximadamente 4 a 6 horas	❑
5. 7 horas ou máis	❑

4.a. ¿Você pratica alguma atividade físicaou esportiva em um clube ou coletivo, sob a direção de um treinador, monitor, instrutor, etc.? (**Considere apenas as atividades que você pratica com uma duração de mais de 30 minutos por dia**)

1. Nunca	❑
2. Menos de uma vez por semana	❑
3. Uma vez por semana	❑
4. 2 a 3 veces por semana	❑
5. 4 a 6 veces por semana	❑
6. Todos os días	❑

4.b. De acordo com o que você disse anteriormente, quanto tempo você diria que você faz esse tipo de atividade física a cada semana com uma intensidade moderada ou alta? (prestes a acabar suando ou cansado)

1. Aproximadamente ½ hora	❑
2. Aproximadamente 1 hora	❑
3. Aproximadamente 2 a 3 horas	❑
4. Aproximadamente 4 a 6 horas	❑
5. 7 horas ou máis	❑

3 – AVALIAÇÃO DA ESCOLA E DA EDUCAÇÃO FÍSICA

5. Em relação à sua vida académica, teve a disciplina de Educação Física? **(<u>Se não teve a disciplina de Educação Física, passe para a questão número 8</u>)**

Sim ❑ Não ❑

6. Tomando como referência a sua experiência pessoal como aluno, como classifica a Educação Física na Escola (ou escolas) que frequentou?

1. Muito má	❑
2. Má	❑
3. Nem má, nem boa	❑
4. Boa	❑
5. Muito boa	❑

7. Declarar a importancia que os factôres explicados abaixo tiveran para a formaciódos tuvieron para a formação de sua opinião.

	Importancia	
	Sim	Não
1. A competência, personalidade e atitude do professor	❑	❑
2. Os benefícios adquiridos (aprendizagem motora, condição física, etc.)	❑	❑
3. Coexistência / relação com colegas	❑	❑
4. Condições materiais / instalações	❑	❑
5. O conteúdo (atividades ensinadas e aprendidas)	❑	❑
6. Organização da Escola / Educação Física	❑	❑
7. As características das classes	❑	❑
8. Outros<br>¿O qué? ____________________________________	❑	❑

14. Relativamente à existência da disciplina de Educação Física no currículo escolar do seu educando, considera que esta disciplina deve **(<u>escolha a afirmação com a qual está mais de acordo</u>)**:

1. Ser obrigatória e os alunos sujeitos a avaliação como nas outras disciplinas	❑
2. Ser obrigatória mas sem que os alunos sejam avaliados	❑
3. Ser opcional	❑
4. Não deve existir	❑
5. Não tenho opinião sobre o assunto	❑

Obrigado pela sua colaboração.
