# Supplementary material for: Parental assessment of physical education in the school curriculum: A brief report on the influence of past experiences as students
Source: PLoS One. 2019 Jul 10;14(7):e0219544. doi: 10.1371/journal.pone.0219544 (PMC6619797; doi:10.1371/journal.pone.0219544)
Supplement: S3 Supporting Information — (PDF) [file pone.0219544.s004.pdf]

UNIVERSIDADE DA CORUÑA

Universidade Vigo

Nº.:

QUESTIONNAIRE FOR PARENTS ABOUT HEALTH, LIFESTYLE AND PHYSICAL EDUCATION

This questionnaire is for a study about Physical Education, health and lifestyle. Therefore, we ask for your cooperation. We appreciate you answering the questions with precision and sincerity. We want to know your opinion.

The questionnaire is anonymous, and the data will be treated with confidentiality. The answers are personal and there is no need to identify yourself. The only identification regards parents and children.

Thank you for covering this questionnaire and for participating in this investigation.

1- PERSONAL INFORMATION

This questionnaire was answered by:

1. Father ❑
2. Mother ❑
3. Other relationship ❑ Which one?____________________

1. Date of birth:

____/____/________
Day Month Year

2. Academic degree and profession:

2.1. Academic degree:

1. Primary education	❑
2. Secondary education	❑
3. Vocational training	❑
4. University level	❑
5. PhD level	❑

2.2. Profession:___________________________________

2 – LIFE HABITS

3.a. How often do you do physical activities/sports **without** being in clubs or associations, such as: walking, running, cycling, swimming, playing football in the street, playing basketball in the park, playing volleyball in the garden or in the beach, etc.? **(Consider only the activities you practice more than 30 minutes per day)**

1. Never	❑
2. Less than once a week	❑
3. Once a week	❑
4. 2 to 3 times a week	❑
5. 4 to 6 times per week	❑
6. Everyday	❑

3.b. According to the previous answer, how long would you say you do this type of physical activity each week with a moderate or high intensity? (about to end up sweating or tired)

1. Approximately ½ hour	❑
2. Approximately 1 hour	❑
3. Approximately 2 to 3 hours	❑
4. Approximately 4 to 6 hours	❑
5. 7 hours or more	❑

4.a. Do you practice any sport/physical activity in a club or association, led by a coach, monitor, instructor, etc.? **(Consider only the activities you practice more than 30 minutes per day)**

1.	Never	❑
2.	Less than once a week	❑
3.	Once a week	❑
4.	2 to 3 times a week	❑
5.	4 to 6 times per week	❑
6.	Everyday	❑

4.b. According to the previous answer, how long would you say you do this type of physical activity each week with a moderate or high intensity? (about to end up sweating or tired)

1.	Approximately ½ hour	❑
2.	Approximately 1 hour	❑
3.	Approximately 2 to 3 hours	❑
4.	Approximately 4 to 6 hours	❑
5.	7 hours or more	❑

3 – OPINION ABOUT THE SCHOOL AND PHYSICAL EDUCATION

5. During your education, did you have the subject Physical Education? **(If you didn't have the subject, go to question number 8)**

Yes ❑ No ❑

6. According to your personal experience as a student, how do you rate the Physical Education in the school you attended?

1. Very bad	❑
2. Bad	❑
3. Neither bad nor good	❑
4. Good	❑
5. Very good	❑

7. Indicate the importance that the factors explained below had for your opinion above.

	Importance	
	Yes	No
1. The competence, personality and attitude of the teacher	❑	❑
2. The acquired benefits (motor learning, physical condition, etc.)	❑	❑
3. Coexistence / relationship with classmates	❑	❑
4. Material conditions / facilities	❑	❑
5. The content (activities taught and learned)	❑	❑
6. Organization of the school / Physical Education	❑	❑
7. The characteristics of the classes	❑	❑
8. Other<br>Which one? ____	❑	❑

8. About the existence of the Physical Education subject in **your child's** school curriculum, I consider that this subject should: **(Choose the affirmation with which you agree the most)**

1. Be compulsory and be evaluated like any other subject	❑
2. Be compulsory without students being evaluated	❑
3. Be optional	❑
4. It should not exist	❑
5. I have no opinion about this matter	❑

Thank you for your cooperation
